# Flavonoids Distinctly Stabilize Lymph Endothelial- or Blood Endothelial Disintegration Induced by Colon Cancer Spheroids SW620

**DOI:** 10.3390/molecules25092066

**Published:** 2020-04-29

**Authors:** Julia Berenda, Claudia Smöch, Christa Stadlbauer, Eva Mittermair, Karin Taxauer, Nicole Huttary, Georg Krupitza, Liselotte Krenn

**Affiliations:** 1 Department of Pharmacognosy, Faculty of Life Sciences, University of Vienna, A-1090 Vienna, Austria; 2 Department of Pathology, Medical University of Vienna, A-1090 Vienna, Austria

**Keywords:** flavonoids, anti-metastatic, structure-activity relationship, colon cancer emboli, endothelial cell micro-environment, 3D co-cultivation

## Abstract

The health effects of plant phenolics in vegetables and other food and the increasing evidence of the preventive potential of flavonoids in “Western Diseases” such as cancer, neurodegenerative diseases and others, have gained enormous interest. This prompted us to investigate the effects of 20 different flavonoids of the groups of flavones, flavonols and flavanones in 3D in vitro systems to determine their ability to inhibit the formation of circular chemorepellent induced defects (CCIDs) in monolayers of lymph- or blood-endothelial cells (LECs, BECs; respectively) by 12(S)-HETE, which is secreted by SW620 colon cancer spheroids. Several compounds reduced the spheroid-induced defects of the endothelial barriers. In the SW620/LEC model, apigenin and luteolin were most active and acacetin, nepetin, wogonin, pinocembrin, chrysin and hispidulin showed weak effects. In the SW620/BEC model acacetin, apigenin, luteolin, wogonin, hispidulin and chrysin exhibited weak activity.

## 1. Introduction

In the metastatic cascade of colon cancer, intravasation into the vasculature is an important early step [1,2], but normally endothelial cells provide resistance against penetrating tumor cells. Invasive tumor cells overcome this hurdle by disintegrating endothelial walls and intravasating the vessel lumen. Therefore, compounds which support the stability of the vasculature may interfere with intravasation and metastasis. Such protective effects could be achieved by inhibition of pro-metastatic metabolite secretion by cancer cells (“key” inhibition) or by abrogating access to the lock in the endothelial barrier (“key-hole” inhibition). In fact, the “key” as well as the “key-hole”, each for itself, are only single components of a considerably complex signaling cascade, both, in cancer cells and in endothelial cells. Hence, blocking respective molecules of the signaling pathways may also prevent metastatic outgrowth. Although potential target molecules have been identified in cancer cells and in endothelial cells, tailored anti-metastatic drugs addressing these targets are currently unavailable. More than 80% of all pharmaceutical drugs in clinical use are derived from or are based on the structures of natural leads [3]. Therefore we started to test extracts of traditional medicinal plants which have been empirically tested in humans over the ages and were selected towards the attenuation of particular ailments with tolerable side effects. This way, we found a methanolic extract of *Scrophularia lucida* L. which affected the intravasation of breast cancer cell spheroids through the lymph endothelial barrier [4]. The promising activity of this extract was based on the flavonoid hispidulin and other unidentified components [5]. However, the concentration which inhibited 50% of circular chemorepellent induced defects (CCIDs) formation (IC_50_) of hispidulin still was to high (~89 µM; [6]) to be considered for therapeutic concepts combating the spreading of breast cancer cells. Here we extended the approach and tested hispidulin and a panel of other structurally related flavonoids in colon cancer models, as orally taken compounds may get directly in contact with the colon epithelium. Therefore, colon cancer cells and their microenvironment, such as blood and lymphatic vasculature, can be treated directly and higher IC_50_ concentrations may be less of a problem for successful interventions. Colon cancer metastasizes to the liver, peritoneum, spinal cord and other more distal organs such as lung, bone etc. [7] through blood vessels and along lymphatic fluids [1,2]. Hence, we established colon cancer intravasation models to investigate molecular mechanisms destabilizing lymph endothelial cell (LEC) barriers and blood endothelial cell (BEC) barriers by colon cancer cell spheroids [8,9] to screen for approved drugs and for natural compounds such as flavonoids [10]. Flavonoids are found in numerous vegetables, spices, fruits and traditional medicinal plants. These compounds were shown to modulate the activity of cancer-related proteins [11] and during the last decades are widely accepted as disease-preventive food components, which seem to be of interest not only in cancer but also in neurodegenerative and cardiovascular disorders [12,13]. Due to the high interest in the scientific community and also in society worldwide, we decided to test 20 flavonoids occurring in medicinal plants (e.g., apigenin and luteolin in chamomile flower or yarrow, acacetin in birch leaf, wogonin and scutellarein in *Scutellaria* species) or in food (e.g., kaempferol in grapes and berries, quercetin in onions, chrysin and pinocembrin in honey). These compounds were studied in two colon cancer intravasation models; the assays consist of SW620 spheroids which are placed on top of either LEC monolayers or BEC monolayers. The arachidonic acid metabolite 12(S)-HETE, which is secreted by SW620 spheroids (the “key”), causes the retraction of LECs or BECs by activating their receptors (the “key holes”). This results in the disintegration of the endothelial barriers [14] and the formation of gaps in the endothelial continuity, which are called circular chemorepellent induced defects (CCIDs). Upon treatment with flavonoids, the inhibition of CCID formation disclosed an improved anti-intravasative activity of some of the tested flavonoids as compared to hispidulin.

## 2. Results

**Figure 1:**
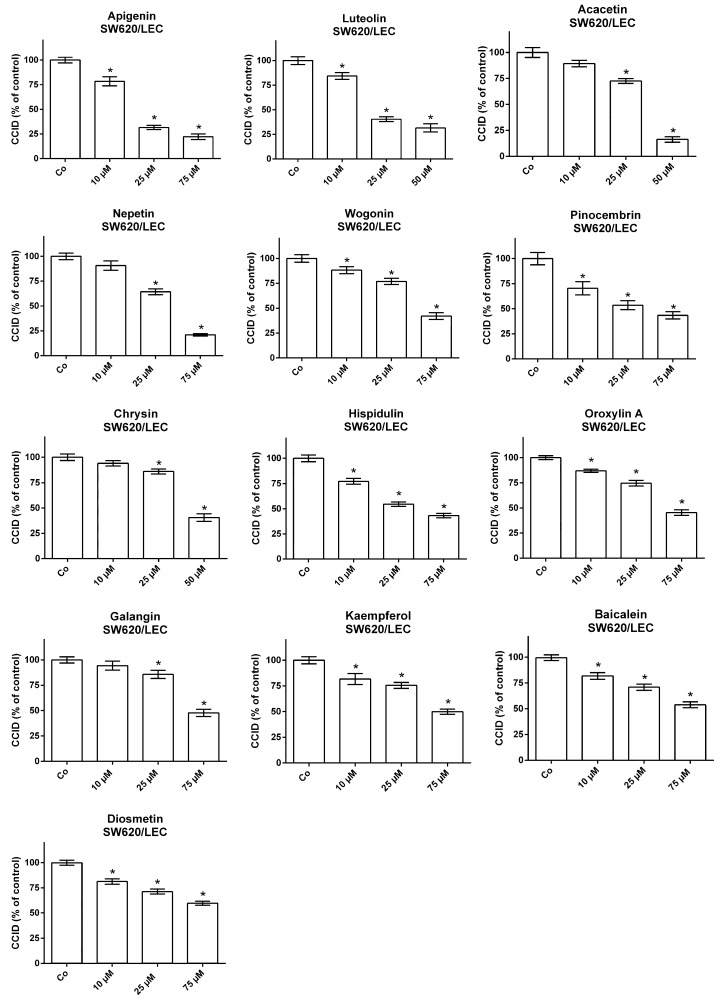
SW620 spheroids were pre-treated for 20 min with solvent (DMSO; Co) or the indicated concentrations of each flavonoid before placement on the lymph endothelial cell (LEC) monolayers and co-cultivation for 4 h. Then circular chemorepellent induced defects (CCIDs) were measured. Three parallel experiments were performed and for each concentration, a total of 15 replicates was analyzed. * *p* < 0.05.

**Figure 2:**
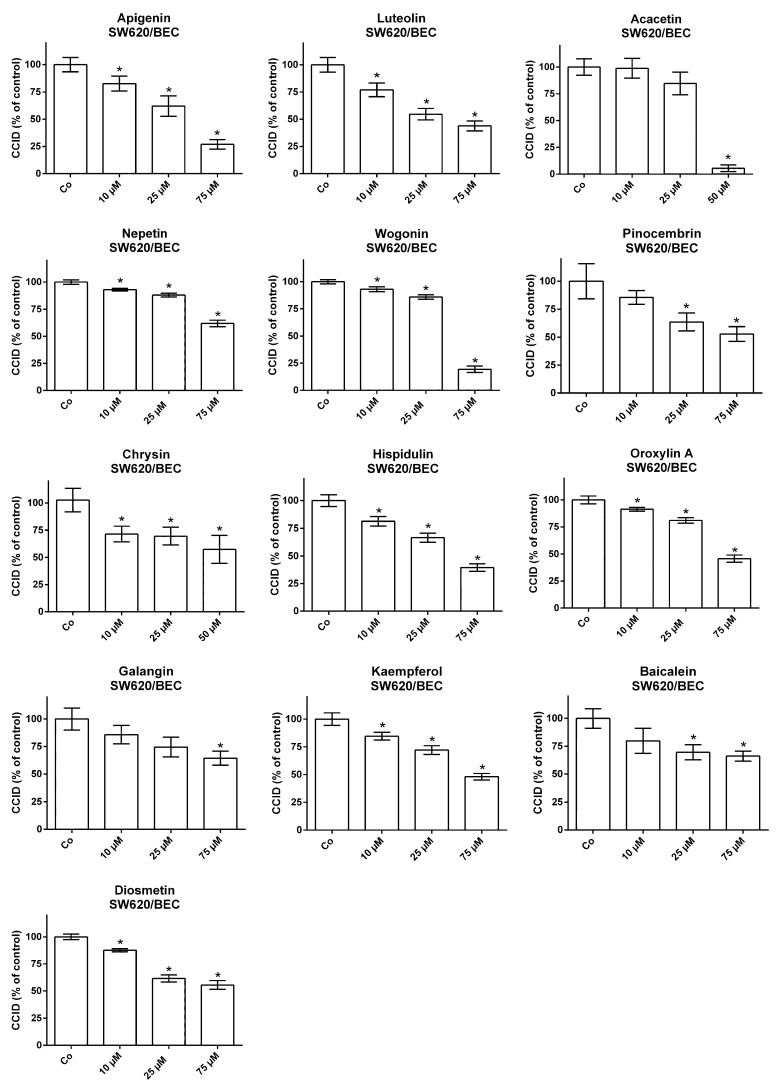
SW620 spheroids were pre-treated for 20 min with solvent (DMSO; Co) or the indicated concentrations of each flavonoid before placement on blood endothelial cell (BEC) monolayers and co-cultivation for 4 h. Then CCIDs were measured. Three parallel experiments were performed. For each concentration, a total of 15 replicates was analyzed. * *p* < 0.05.

**Figure 3:**
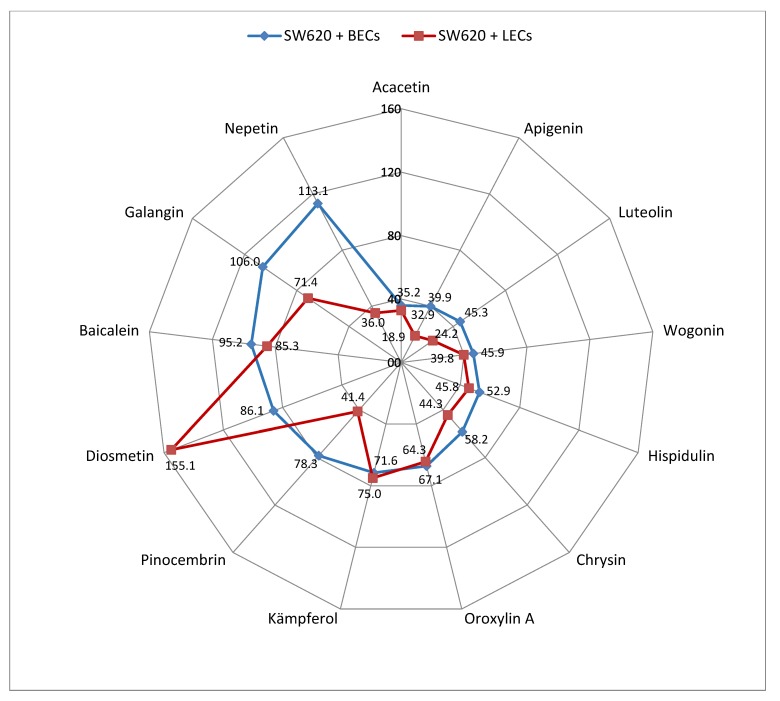
Inhibition of of CCID formation (IC_50_) values; µM) by studied flavonoids in the SW620/BEC (blue line) and the SW620/LEC model (red line) with at least one of them <90 µM. The results are the mean pooled from 3 independent experiments performed in 5-fold measurements (*n* = 3).

SW620 spheroids were placed on top of either LEC- or BEC-monolayers, which were treated with increasing concentrations of the individual flavonoids (Figure 1 and Figure 2). In general, the SW620/LEC model was more sensitive to flavonoid-mediated inhibition of CCID formation than the SW620/BEC model. We categorized flavonoids, which inhibited 50% of CCID formation (IC_50_), at concentrations up to 30 µM as active, up to 60 µM as weakly active and above 60 µM as inactive. In the LEC model apigenin and luteolin were active with IC_50_ values of 18.9 and 24.2 µM, respectively. Acacetin, nepetin, wogonin, pinocembrin, chrysin and hispidulin showed weaker activities (IC_50_ between 32.9 and 45.7 µM; Figure 3, Supplemental Table S1). In the BEC model the activity was generally lower and acacetin, apigenin, luteolin, wogonin, hispidulin and chrysin were weakly active (Figure 3, Supplemental Table S1). The other flavonoids (oroxylin, galangin, kaempferol, baicalein, eriodictyol, homoeriodyctiol, diosmetin, scutellarin, herbacetin, naringenin, quercetin and norwogonin in the LEC model; oroxylin, kaempferol, scutellarin, pinocembrin, diosmetin, baicalein, galangin, nepetin, homoeriodictyol, eriodyctiol, herbacetin, naringenin, quercetin and norwogonin in the BEC model; Supplemental Table S1) were considered inactive.

All six compounds, which were at least weakly active in both models (apigenin, luteolin, acacetin, wogonin, chrysin and hispidulin) are members of the class of flavones. In addition, chrysin, apigenin, luteolin and acacetin share the structural similarity of a lack of OH-groups at positions 6 and 8 (Supplemental Table S1). An OH group at position 4´ (apigenin), OH groups at positions 3´ and 4´ (luteolin), or an OCH_3_ group at position 4´ (acacetin) increased the activity in the SW620/LEC model 2.3-, 1.8-, and 1.3-fold, respectively, when compared to chrysin. In the SW620/BEC model the activity increased 1.5-, 1.3-, and 1.6-fold.

Attachment of an additional OCH_3_ group at position 8 in wogonin increased the activity compared to chrysin by 1.1- and 1.3-fold in the LEC and BEC model, respectively (Supplemental Table S1). Comparison of hispidulin which differs by an additional OCH_3_ group at position 6 from apigenin showed a reduction of the activity in the LEC model, but less difference in the BEC model. The substitution of hispidulin did not influence the activity compared to chrysin in both models (Supplemental Table S1).

A converse trend in the case of a substitution with an OCH_3_ group in position 6 was observed when comparing nepetin (= 6-methoxy-luteolin) with luteolin: The additional OCH_3_ group abrogated the CCID-inhibitory effect of nepetin in the BEC model, but resulted in only minor changes in the LEC model. The activity of nepetin was 3.1-fold higher in the LEC model compared to the BEC model (Supplemental Table S1). However, it was not possible to determine which of the substituents were responsible for the specificity towards the LEC model.

In an earlier study, flavanones remained without any effects in a similar CCID model for breast cancer based on MCF7/LEC cells [6]. Surprisingly, in the here presented colon cancer models, the flavanone pinocembrin was weakly active in the LEC model and, although categorized as inactive, showed an IC_50_ of 78.3 µM in the BEC model (Supplemental Table S1).

The 1.9-fold gain of activity of pinocembrin in the LEC model compared to the BEC model was similar to the differences observed for luteolin and apigenin in the two models. Nevertheless, the mechanisms responsible for these differences could not be elucidated.

Based on these results, the following structural characteristics determined the general activity in both models:

Only flavones with the typical 2-phenyl-5,7-dihydroxy-chromen-4-one structure showed activity in both models. The substitution pattern in ring B has minor effects: an OH- or OCH_3_-group in position 4’ as well as two OH-groups in position 3’ and 4’ resulted in comparable activity for apigenin, acacetin and luteolin, respectively (Supplemental Table S1). The lack of any OH- or OCH_3_-group in ring B (chrysin) led to slightly lower activity. In diosmetin, with a 3’ OH- and 4’ OCH_3_-group, the methylation of 4’-OH resulted in a complete loss of the activity. Additional OH-groups in ring A at positions 6 or 8, respectively, also resulted in such a loss as shown for scutellarein, baicalein and norwogonin. Replacement of the 6- or 8-OH group by a 6- or 8-OCH_3_-group in the more apolar compounds hispidulin and oroxylin A or wogonin, respectively, moderately increased the activity in both models, whereas the 6’-OCH_3_-group in nepetin led to a significant rise in the effect only in the LEC model. All flavonols (derivatives of 2-phenyl-3,5,7-trihydroxy-chromen-4-one, namely galangin, kaempferol, herbacetin, and quercetin) remained without effects in both models. This confirmed the assumption that hydroxylation in position 3 is suppressing the CCID inhibitory activity.

From the four tested flavanones lacking the 2,3 double bond, only pinocembrin showed moderate activity in the LEC model whereas no activity was seen for naringenin, eriodictyol and homoeriodictyol.

No correlation between the activities in both CCID models and the logP values of the compounds was seen [6].

## 3. Discussion

Intravasation is a very early step in metastasis and its prevention is expected to attenuate cancer progression and the associated fatalities. Colon cancer metastasizes to distant organs by intravasating blood as well as lymph endothelial vessels [7], which is different to the spreading of breast cancer cells, which may prefer to spread along the lymphatic route [15,16]. The colon epithelium and also colon cancer emboli are directly exposed to bypassing colon content and therefore directly accessible to treatment via oral (or anal) intake. Flavonoids are contained in various foods and show anti-proliferative effects in vitro [17] and anti-carcinogenic activities in humans [18]. Therefore, these natural compounds have gained much interest as preventive or therapeutic agents [19,20] and, in fact, the risk of colorectal cancer was significantly reduced upon high consumption of anthocyanidins, flavan-3-ols and flavonols [21]. This motivated us to study the anti-intravasative properties of 20 flavonoids, which were screened in 3D colon cancer in vitro intravasation models consisting of SW620 colon cancer cell spheroids and BEC or LEC monolayers. The analyses of the disruption of blood and lymph endothelial barriers as well as the inhibition of disruption by these compounds should provide new insights into structure–activity relations.

Consistent with earlier results in a MCF7/LEC model [6], the 2-phenyl-5,7-dihydroxy-chromen-4-one structure of flavones without any additional substitution in positions 6 or 8 seems essential for the effects in all three 3D models and the IC_50_ values of the most active substances apigenin, acacetin, luteolin and chrysin were in a comparable dimension.

Most of the compounds with additional OH-groups in positions 3, 6 and 8 such as baicalein, norwogonin, galangin, scutellarein or herbacetin showed no activity in all assays independent of the epithelial as well as the cancer cell lines used [6]. Substitution with more apolar OCH_3_-groups in positions 6 or 8 e.g., in hispidulin or oroxylin A resulted in weak and almost weak activity, respectively, in both assay models. The weak activity of hispidulin was specific for both SW620/EC models, because it had been inactive in the MCF7/LEC model. In addition, the OCH_3_ group at position 8 of wogonin conferred specificity to the SW620/EC models as it had been inactive in the MCF7/LEC model [6].

Comparison of the CCID inhibitory effects in both SW620 models versus the MCF7/LEC model [6] confirmed that flavanones obviously are not able to suppress the disruption of the endothelial barriers in the CCID assay. Pinocembrin was the only exemption of four compounds from this class and showed a moderate inhibition in the SW620/LEC model.

Although immediate contact of flavonoids with cancer emboli within the colon epithelium and their microenvironment may allow higher IC_50_ values, the concentrations of these compounds certainly remain an issue. To this end appropriate formulations for apigenin [22] and luteolin [23] resulted in plasma concentrations at which, in our study, significant effects were achieved and the oral intake of 50 mg/kg/day chrysin reduced lung metastases in an experimental breast cancer model [24]. However, caution has to be taken as a recent in vitro study showed that low concentrations (1 µM and less) of acacetin seem to promote MCF-7 breast cancer cell growth rather than to inhibit it [25].

Interestingly, nepetin (=eupafolin) seemed to interfere specifically with the cancer cell–lymph endothelial interaction, because it inhibited the SW620 intravasation through LECs, but not through BECs. The nepetin-specific interaction between SW620 and LECs was obviously due to the 3´OH group, because hispidulin (no 3´OH group) was almost similarly active in both models. A nepetin-nanoparticle delivery system achieved high concentrations which were sufficient to attenuate LPS-induced renal damage in vivo [26]. In mouse experiments, human esophagus cancer xeno-transplants were inhibited by nepetin (20 mg/kg, 3x per week, i.p.) [27], and a similar activity for nepetin was observed in HEPG2 and HEPG3 xenografts (60 mg/kg, 3x per week, i.p.) [28]. Specifically, nepetin reduced the migration and tube formation of HUVECs and the neo-angiogenesis in xenograft tumors [28], and furthermore, nepetin inhibited ICAM-1 expression through nuclear factor-kappa B (NF-kB) in A549 human lung airway epithelial cells [29]. We have demonstrated that the adhesion of breast cancer cells and LECs, similar to the adhesion of colon cancer cells and LECs [30], is mandatory for intravasation and depends on ICAM-1 in LECs [31,32]. Therefore, in a scenario in which lymphatic dissemination of cancer cells is an issue, the detailed study of the inhibitory mechanisms of nepetin may provide insights for more specific treatment options. It is widely accepted that breast cancer cells spread through the lymphatic vasculature and also colon cancer cells colonize, apart from liver, various organs along the lymphatic route [1,2]. For these cancer entities nepetin may specifically attenuate their dissemination.

Among the many molecular pathways in the different steps of cancer progression, the inhibition of NF-kB is well known as a very important step in tumorigenesis and metastasis and natural polyphenols were shown to act on this mechanism [33]. In earlier studies we have shown that it is relevant for the retraction and CCID formation of LECs as well [31,32]. Several flavonoids in this study, namely apigenin [34], acacetin [35], luteolin [36], chrysin [37], hispidulin [38], wogonin [39], nepetin [40] and pinocembrin [41] inhibit the NF-kB pathway. Numerous investigations also revealed that the proper function of BECs relies on this pathway [42,43,44]. Thus, we hypothesize that the inhibition of NF-kB is the most important mechanism of the flavonoids attenuating the formation of CCIDs.

## 4. Materials and Methods

### 4.1. Chemicals

The compounds kaempferol, acacetin, galangin, chrysin, homoeriodictyol were obtained from Carl Roth GmbH (Karlsruhe, Germany), apigenin, nargingenin, hispidulin, scutellarein, baicalein, quercetin, wogonin, and diosmetin from Sigma-Aldrich (Vienna, Austria), herbacetin, pinocembrin, luteolin, eriodictyol, norwogonin from Extrasynthése (Genay, France) and nepetin as well as oroxylin A from Phytolab (Vestenbergsgreuth, Germany). All stock solutions were prepared in dimethylsulfoxide (DMSO; 99.7% cell culture grade, Sigma-Aldrich (Vienna, Austria).

### 4.2. Cell Culture

Human dermal microvascular endothelial cells (Clonetics, Basel, Switzerland) were telomerase-immortalized and lymphatic- and blood- endothelial cells were prepared as described [45,46] and cultivated in microvascular endothelial cell growth medium-2 (EGM2 MV; Clonetics CC-4147; Lonza Group Ltd., Basel, Switzerland). The SW620 (CCL-227TTM) cell line was obtained from the American Type Culture Collection (ATCC; Rockville, MD, USA) and grown in RPMI-1640 medium supplemented with 10% fetal calf serum and 1% penicillin/streptomycin. All cells were maintained in humidified atmosphere containing 5% CO_2_ at 37 °C. All media were provided by Life Technologies (Lofer, Austria).

#### 4.2.1. SW620 Spheroid Generation

SW620 cells were transferred to 30 mL DMEM medium with 6 mL of a 1.6% methylcellulose solution (0.3% final concentration; Cat. No.: M-512, 4000 centipoises; Sigma, Munich, Germany). The cell suspension (150 μL) was transferred to each well of a 96-well round bottom plate (Greiner Bio-one, Cellstar 650185, Kremsmünster, Austria) and centrifuged at 1000 rpm for spheroid formation within the following four days. This resulted in sufficient density of the spheroids for further manipulations.

#### 4.2.2. 3-D Co-Cultivation of SW620 Colon Cancer Cells with LECs and BECs and Circular Chemorepellent Induced Defect (CCID) Assay

SW620 spheroids (2000 cells/spheroid) were washed with DPBS. Solutions of the different flavonoids (10, 25 and 50 or 75 µM in EGM) were prepared and the spheroids were transferred into these solutions. After 30 min of incubation at 37 °C at least 12 spheroids per well were placed on the cytotracker-stained LEC or BEC monolayers that had been seeded into 24 well plates (Costar 3524, Sigma, Munich, Germany) in 2 mL EGM2 MV medium [10,34].

**Figure 4:**
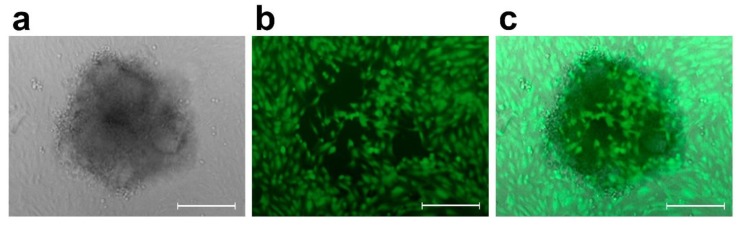
CCID assay set-up. (**a**) A SW620 cell spheroid (phase contrast microscopy) placed on top of (**b**) a monolayer of LECs stained with cytotracker (green; fluorescence microscopy using a FITC filter). After 4 h co-cultivation, the developed CCID in the LEC-monolayer due to 12(S)-HETE secreted by the spheroid, which causes the retraction of LECs, was photographed with 200-fold magnification. (**c**) Merge of phase contrast and fluorescence micrography. Scale bars: 200 µM.

**Figure 5:**
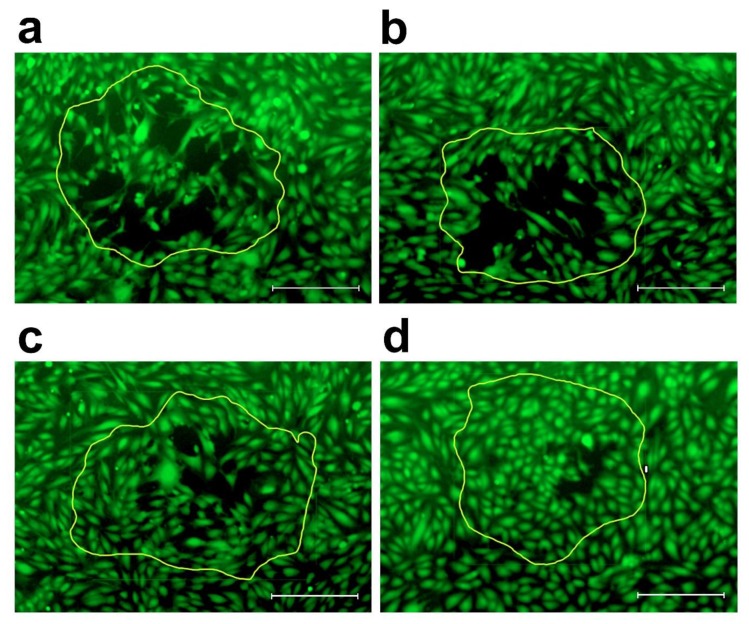
Inhibition of the formation of CCIDs by increasing concentrations of apigenin. SW620 cell spheroids and lymph endothelial cell monolayers (LECs) were pre-incubated either with (**a**) solvent (CO; DMSO), (**b**) 10 µM apigenin, (**c**) 25 µM apigenin and (**d**) 75 µM apigenin for 20 min. Then, apigenin pre-treated SW620 spheroids were placed on top of apigenin pre-treated LECs and co-cultivated for 4 h. Subsequently, the development of CCIDs within the LEC-layer was photographed. The bright lines contour the positions of the spheroids. Scale bars: 200 µM.

After four hours of incubation of the SW620 spheroids-LEC monolayer co-cultures or 3 h in case of SW620 spheroids-BEC monolayer co-cultures, the CCIDs in the LEC or BEC monolayer underneath the spheroids were photographed with an Axiovert (Zeiss, Jena, Germany) fluorescence microscope to visualize cytotracker (green)-stained LECs or BECs underneath the spheroids (Figure 4 and Figure 5) [10,34].

Gap areas were calculated with the Axiovision Re. 4.5 6 software (Carl Zeiss, Jena, Germany). SW620 spheroids treated with solvent (DMSO) served as negative control. The gap sizes of at least 12 spheroids per experiment were measured.

### 4.3. Statistical Analysis

Statistics were determined using Student’s t test, analysis of variance (ANOVA) or nonlinear regression as appropriate calculated by Graph Pad Prism 5.0 software. Significance was designated as * for *p* < 0.05. Error bars depict ± SD for *n* = 3.

## 5. Conclusions

The potential of flavonoids to avoid the disintegration of LEC or BEC barriers by SW620 colon cancer spheroids, which are a model of the first steps of metastasis, clearly depended on the flavone substructure (a 2,3 double bond) and the grade of hydroxylation. This was confirmed by the highest activity for flavones with moderate hydroxylation. These results for the first time show the activity of 20 flavonoids in the CCID assay with SW620 spheroids. We were able to confirm data which have proven the importance of the 2,3 double bond in ring C of the flavonoid scaffold for an activity on early mechanisms in the progression of cancer. A sufficient supply of the most active compounds might be achieved via special delivery systems.

## Supplementary Materials

The following are available online. Table S1: IC_50_ values and 95% CI of 20 tested flavonoids in the BEC and the LEC model.
