# Supplementary material for: Flavonoids Distinctly Stabilize Lymph Endothelial- or Blood Endothelial Disintegration Induced by Colon Cancer Spheroids SW620
Source: Molecules. 2020 Apr 29;25(9):2066. doi: 10.3390/molecules25092066 (PMC7248751; doi:10.3390/molecules25092066)
Supplement: Supplementary file 1 [file molecules-25-02066-s001.pdf]

Table S1: IC_{50} values and 95% CI of 20 tested flavonoids in the BEC and the LEC model

Flavanones							
No.	Compound	Formula	IC_{50} BEC model	95% CI	IC_{50} LEC model	95% CI	logP (o/w) [1]
**1**	Pinocembrin	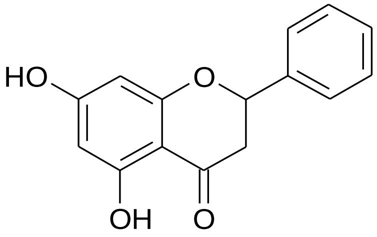	78.3 µM	57.3-110.3	41.4 µM	32.6-53.4	2.69
**2**	Naringenin	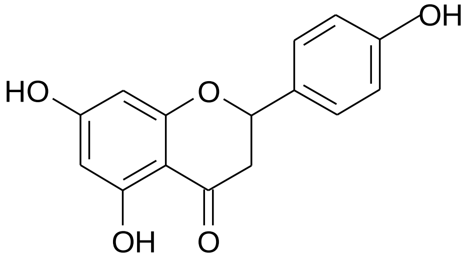	> 200 µM	---	> 200 µM	---	2.38
**3**	Eriodictyol	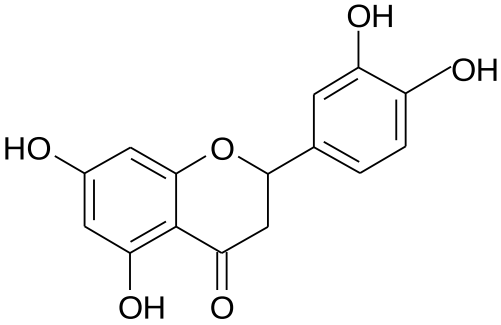	> 100 µM	---	94.8 µM	---	2.11

Table S1 continued

No.	Compound	Formula	IC_{50} BEC model	95% CI	IC_{50} LEC model	95% CI	logP (o/w) [1]
**4**	Homoeriodictyol	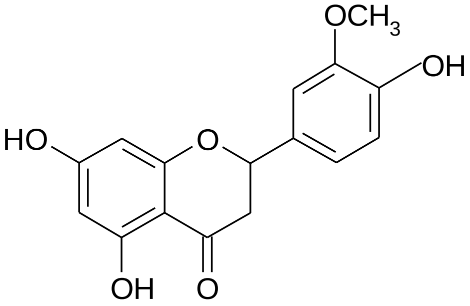	> 100 μM	---	> 100 μM	---	2.37

Table S1 continued

Flavones and flavonols							
No.	Compound	Formula	IC_{50} BEC model	95% CI	IC_{50} LEC model	95% CI	logP (o/w)[1]
**5**	Chrysin	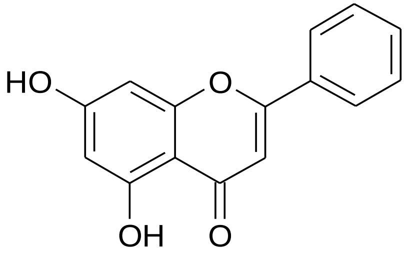	58.2 μM	31.4-97.0	44.3 μM	31.7-60.4	2.84
**6**	Galangin	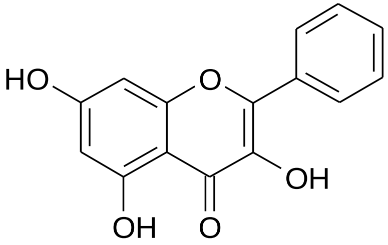	> 100 μM	---	71.4 μM	58.1-92.4	2.61
**7**	Baicalein	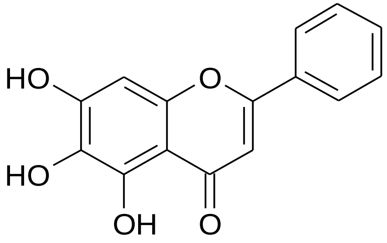	95.2 μM	---	85.3 μM	73.8-99.1	2.49

Table S1 continued

No.	Compound	Formula	IC_{50} BEC model	95% CI	IC_{50} LEC model	95% CI	logP (o/w)[1]
**8**	Oroxylin A	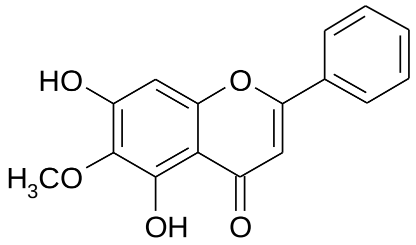	67.1 µM	58.8-82.9	64.3 µM	55.1-72.4	2.76
**9**	Norwogonin	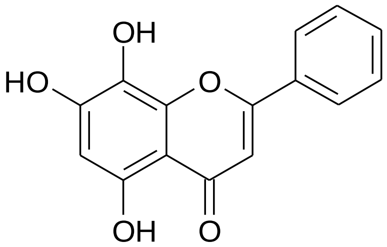	> 200 µM	---	> 200 µM	---	2.49
**10**	Wogonin	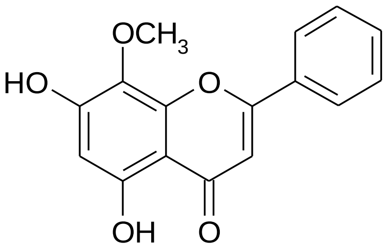	45.9 µM	36.5-67.9	59.6µM	48.5-70.0	2.76
**11**	Apigenin	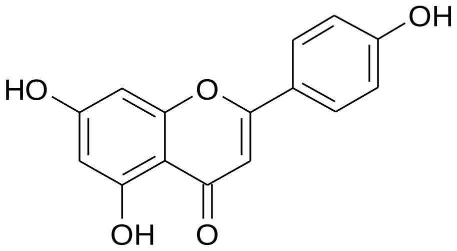	39.9 µM	25.0-53.3	18.9 µM	14.9-23.9	2.53

Table S1 continued

No.	Compound	Formula	IC_{50} BEC model	95% CI	IC_{50} LEC model	95% CI	logP (o/w)[1]
**12**	Acacetin	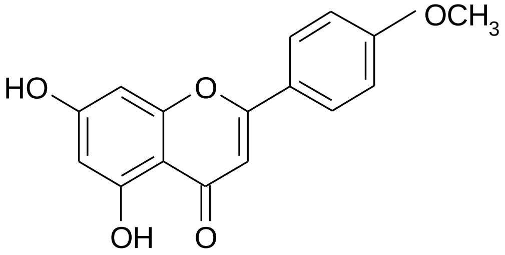	36,0 µM	18.7-51.6	32,9 µM	24.9-42.6	2.80
**13**	Kämpferol	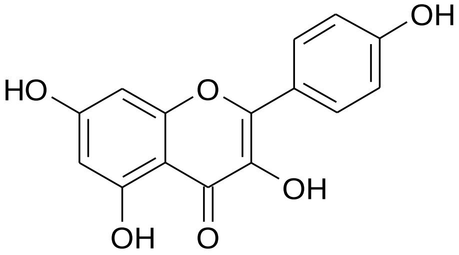	71.5 µM	59.9-85.6	75.0 µM	55.5-90.0	2.31
**14**	Scutellarein	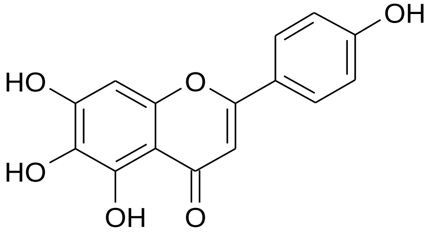	71.7 µM	59.1-87.8	> 200 µM	---	2.19
**15**	Herbacetin	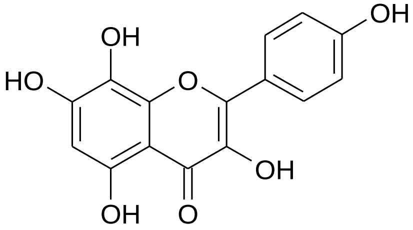	> 200 µM	---	> 200 µM	---	1.96

Table S1 continued

No.	Compound	Formula	IC_{50} BEC model	95% CI	IC_{50} LEC model	95% CI	logP (o/w)[1]
**16**	Hispidulin	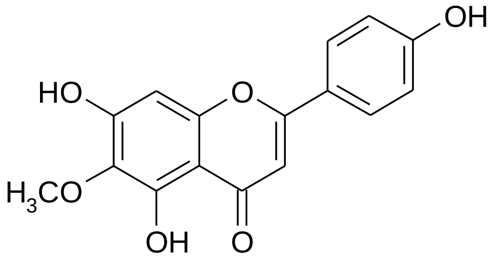	52.9 µM	43.3-59.4	45.7 µM	39.9-53.2	2.45
**17**	Luteolin	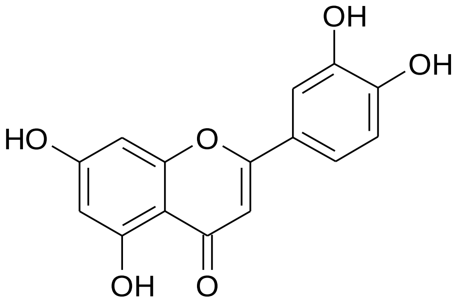	45.3 µM	34.6-59.9	24.2 µM	18.8-29.4	2.26
**18**	Diosmetin	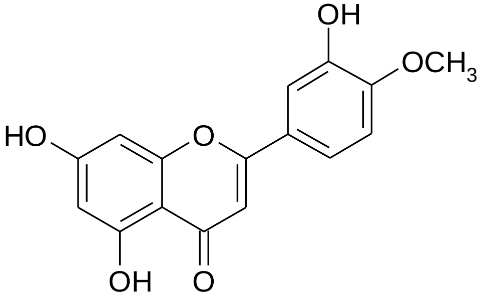	86.1 µM	73.3-102.0	> 100 µM	---	2.31

Table S1 continued

No.	Compound	Formula	IC_{50} BEC model	95% CI	IC_{50} LEC model	95% CI	logP (o/w)[1]
**19**	Nepetin	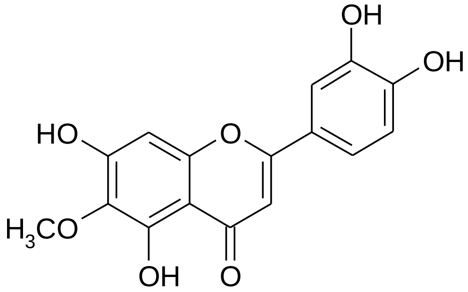	> 100 µM	---	35.2 µM	28.6-42.7	2.18
**20**	Quercetin	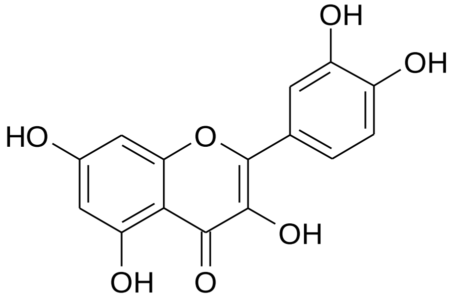	> 200 µM	---	> 200 µM	---	2.03

> 100 µM = extrapolated IC_{50} values between 100 and 200 µM;

[1] Eichsteininger, J.; Kirisits, K.; Smöch, C.; Stadlbauer, C.; Nguyen, C. H.; Jäger, W.; Özmen, A.; Ecker, G.; Krupitza, G.; Krenn, L. Structural Insight into the In Vitro Anti-Intravasative Properties of Flavonoids. *Sci Pharm* **2019,** *87*, 23. https://doi.org/10.3390/scipharm87030023
